# Supplementary material for: Incorporating strontium enriched amorphous calcium phosphate granules in collagen/collagen-magnesium-hydroxyapatite osteochondral scaffolds improves subchondral bone repair
Source: Mater Today Bio. 2024 Jan 20;25:100959. doi: 10.1016/j.mtbio.2024.100959 (PMC10847994; doi:10.1016/j.mtbio.2024.100959)
Supplement: Multimedia component 1 [file mmc1.docx]

**Supplementary Material**

**Table S1:** Clinical Orthopedic Assessment. This tool is to evaluate the clinical health of goat joints.

| **Parameter** | **Variables** | **Score** |
| --- | --- | --- |
| Lameness | Walks normally | 5 |
|  | Slightly lame when walking | 4 |
|  | Moderately lame when walking | 3 |
|  | Severely lame when walking | 2 |
|  | Reluctant to rise and will not walk more than five paces | 1 |
| Joint mobility | Full range of motion | 5 |
|  | Mild limitation (10–20%) in ROM; no crepitus | 4 |
|  | Mild limitation (10–20%) in ROM; with crepitus | 3 |
|  | Moderate limitation (20–50%) in ROM; ±crepitus | 2 |
|  | Severe limitation (>50%) in ROM; ±crepitus | 1 |
| Pain on knee palpation and movement | None | 5 |
|  | Mild signs; Goat turns head in recognition | 4 |
|  | Moderate signs; Goat pulls limb away | 3 |
|  | Severe signs; Goat vocalises or becomes aggressive | 2 |
|  | Goat will not allow palpation | 1 |
| Weight-bearing | Equal on all limbs standing and walking | 5 |
|  | Normal standing; favours affected limb when walking | 4 |
|  | Partial weight-bearing standing and walking | 3 |
|  | Part. weight-bearing standing; non-weight-bearing walk | 2 |
|  | Non-weight-bearing standing and walking | 1 |
| Overall score of clinical condition | Not affected | 5 |
|  | Mildly affected | 4 |
|  | Moderately affected | 3 |
|  | Severely affected | 2 |
|  | Very severely affected | 1 |
| Total score |  | 25 |

**Table S2:** Macroscopic joint Assessment. This tool is to evaluate the macroscopic normalization of goat joints when the joints were opened.

| **Parameter** | **Variables** | **Score** |
| --- | --- | --- |
| Wound healing abnormal | Yes | 0 |
|  | No | 1 |
| Swelling of soft tissues surrounding joints | Yes | 0 |
|  | No | 1 |
| Effusion of the joints | Yes | 0 |
|  | No | 1 |
| Patellar luxation | Yes | 0 |
|  | No | 1 |
| Joint mobility abnormal | Yes | 0 |
|  | No | 1 |
| Adhesions in the joint | Yes | 0 |
|  | No | 1 |
| Erosions of the joint | Yes | 0 |
|  | No | 1 |
| Synovial fluid abnormal | Yes | 0 |
|  | No | 1 |
| Synovial membrane abnormal | Yes | 0 |
|  | No | 1 |
| Lesion on the opposite cartilage surface  (trochlear groove vs patella) | Yes | 0 |
|  | No | 1 |
| Lesion on the opposite cartilage surface  (medial femoral condyle vs meniscus/tibia plateau) | Yes | 0 |
|  | No | 1 |
|  | **Total** | **0-11** |

**Table S3:** International Cartilage Repair Society (ICRS) cartilage repair scoring system. This tool is to evaluate the macroscopic appearance of cartilage repair tissue.

| **Parameter** | **Variables** | **Scores** |
| --- | --- | --- |
| Degree of defect repair | In level with surrounding cartilage | 4 |
|  | 75% repair of defect depth | 3 |
|  | 50% repair of defect depth | 2 |
|  | 25% repair of defect depth | 1 |
|  | 0% repair of defect depth | 0 |
| Integration to border zone | Complete integration with surrounding cartilage | 4 |
|  | Demarcating border < 1 mm | 3 |
|  | ¾ of graft integrated, ¼ with a notable border > 1 mm | 2 |
|  | 1/2 of graft integrated with surrounding cartilage,1/2 with a notable border > 1 mm | 1 |
|  | From no contact to ¼ of graft integrated with surrounding cartilage | 0 |
| Macroscopic appearance | Intact smooth surface | 4 |
|  | Fibrillated surface | 3 |
|  | Small, scattered fissures or cracks | 2 |
|  | Several, small or few but large fissures | 1 |
|  | Total degeneration of grafted area | 0 |
| **Overall** | Grade I normal  Grade II nearly normal  Grade III abnormal  Grade IV severely abnormal | 12  11-8  7-4  3-1 |

**Table S4:** A semi-quantitative macroscopic scoring system developed by Goebel et al. for the macroscopic description of articular cartilage repair.

| **Parameter** | **Variables** | **Scores** |
| --- | --- | --- |
| Color of the repair tissue | Hyaline or white | 4 |
|  | Predominantly white (>50%) | 3 |
|  | Predominantly translucent (>50%) | 2 |
|  | Translucent | 1 |
|  | No repair tissue | 0 |
| Presence of blood vessels in the repair tissue | No | 4 |
|  | Less than 25% of the repair tissue | 3 |
|  | 25-50% of the repair tissue | 2 |
|  | 50-75% of the repair tissue | 1 |
|  | More than 75% of the repair tissue | 0 |
| Degeneration of adjacent articular cartilage | Normal | 4 |
|  | Cracks and/or fibrillations in integration zone | 3 |
|  | Diffuse osteoarthritic changes | 2 |
|  | Extension of defect into the adjacent cartilage | 1 |
|  | Subchondral bone damage | 0 |
| Surface of the repair tissue | Smooth, homogeneous | 4 |
|  | Smooth, heterogeneous | 3 |
|  | Fibrillated | 2 |
|  | Incomplete new repair tissue (rough) | 1 |
|  | No repair tissue | 0 |
| Percentage defect filling | 80-100 % | 4 |
|  | 60-80 % | 3 |
|  | 40-60 % | 2 |
|  | 20-40% | 1 |
|  | 0-20 % | 0 |
| **Total Scores** |  | **20** |


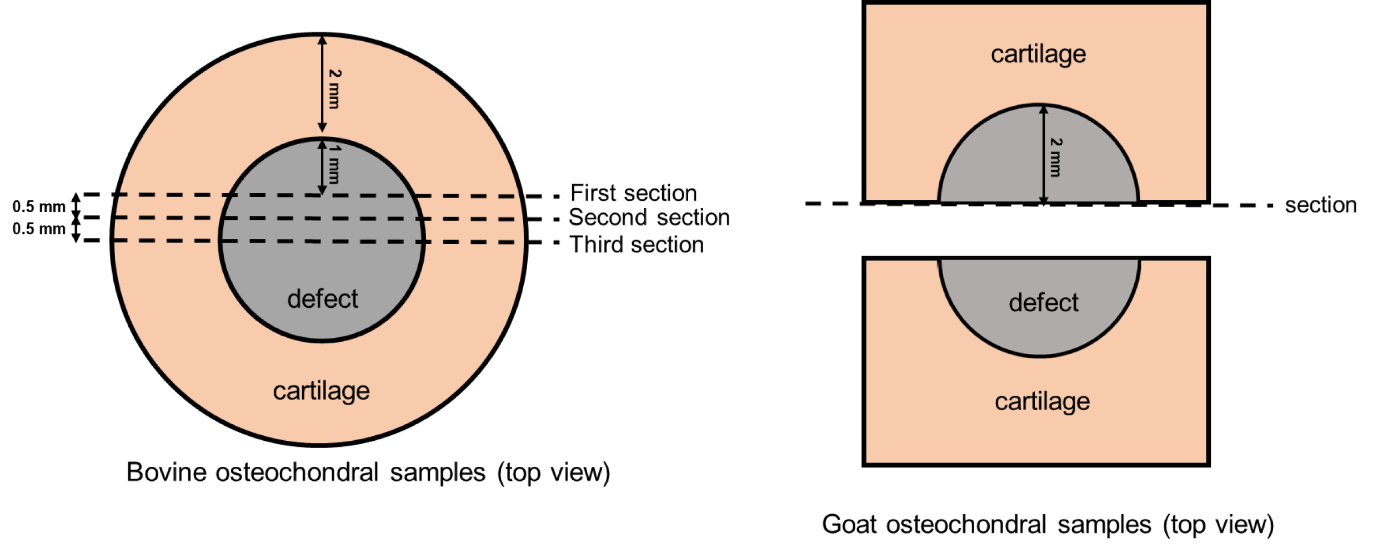


Figure S1. Collection of sections from mouse model and goat model for histology.


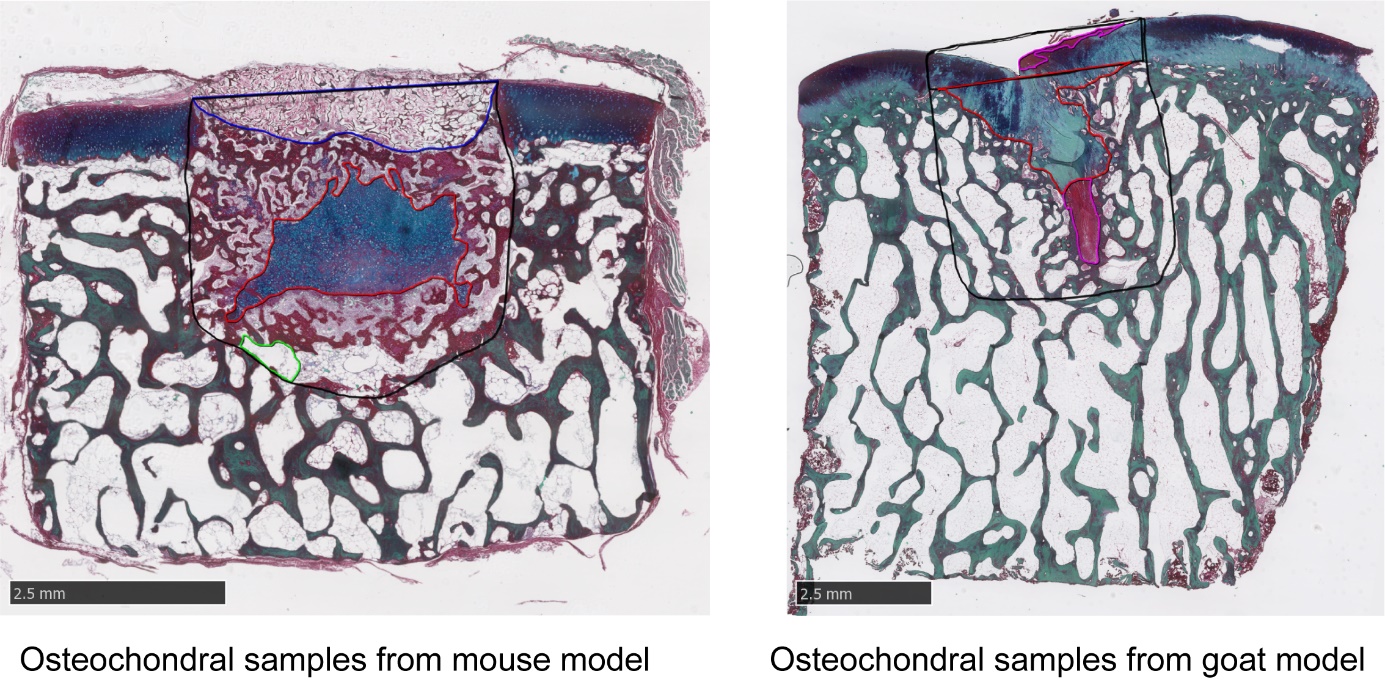


Figure S2. Example on defining the defect region, newly formed cartilage-like tissue formation, bone-like tissue formation, fibrous-like tissue formation, remnants of the scaffold for quantification. Scale bars indicated 2.5 mm.


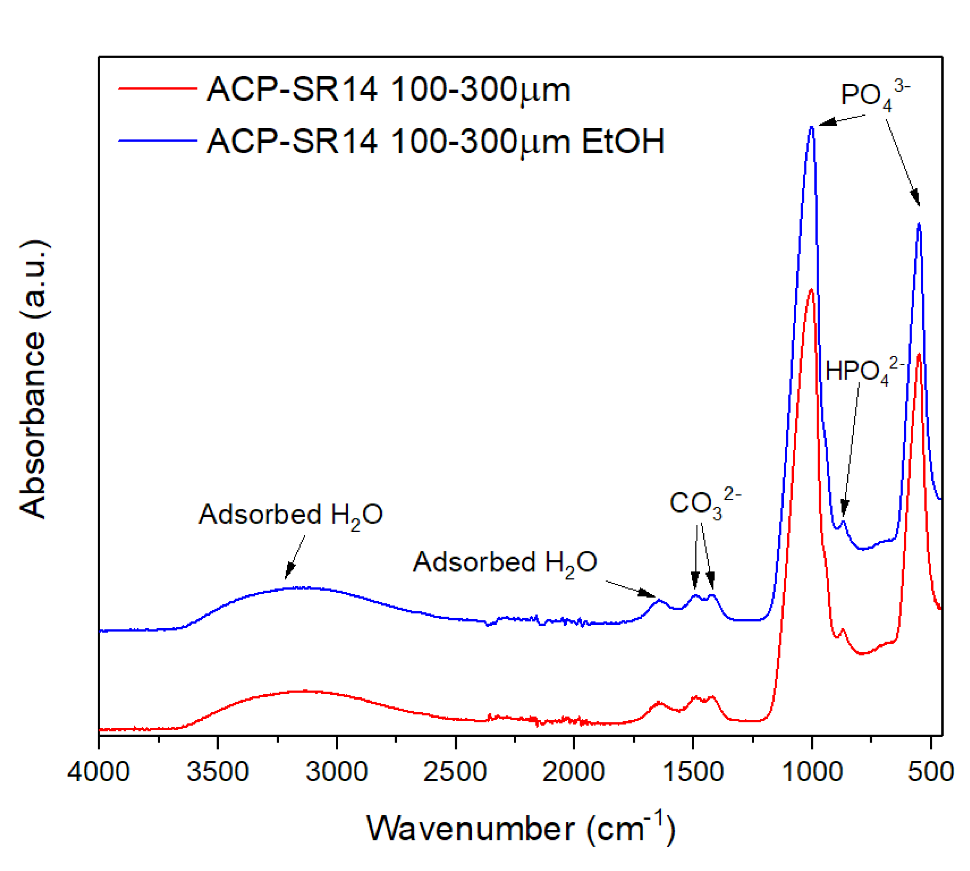


Figure S3: FT-IR spectra of ACP granules before and after rinsing in EtOH to remove debris resulted from dry milling process.

Figure S4: XRD patterns (A) and (FT-IR) spectra of ACP granules before and after γ sterilization.


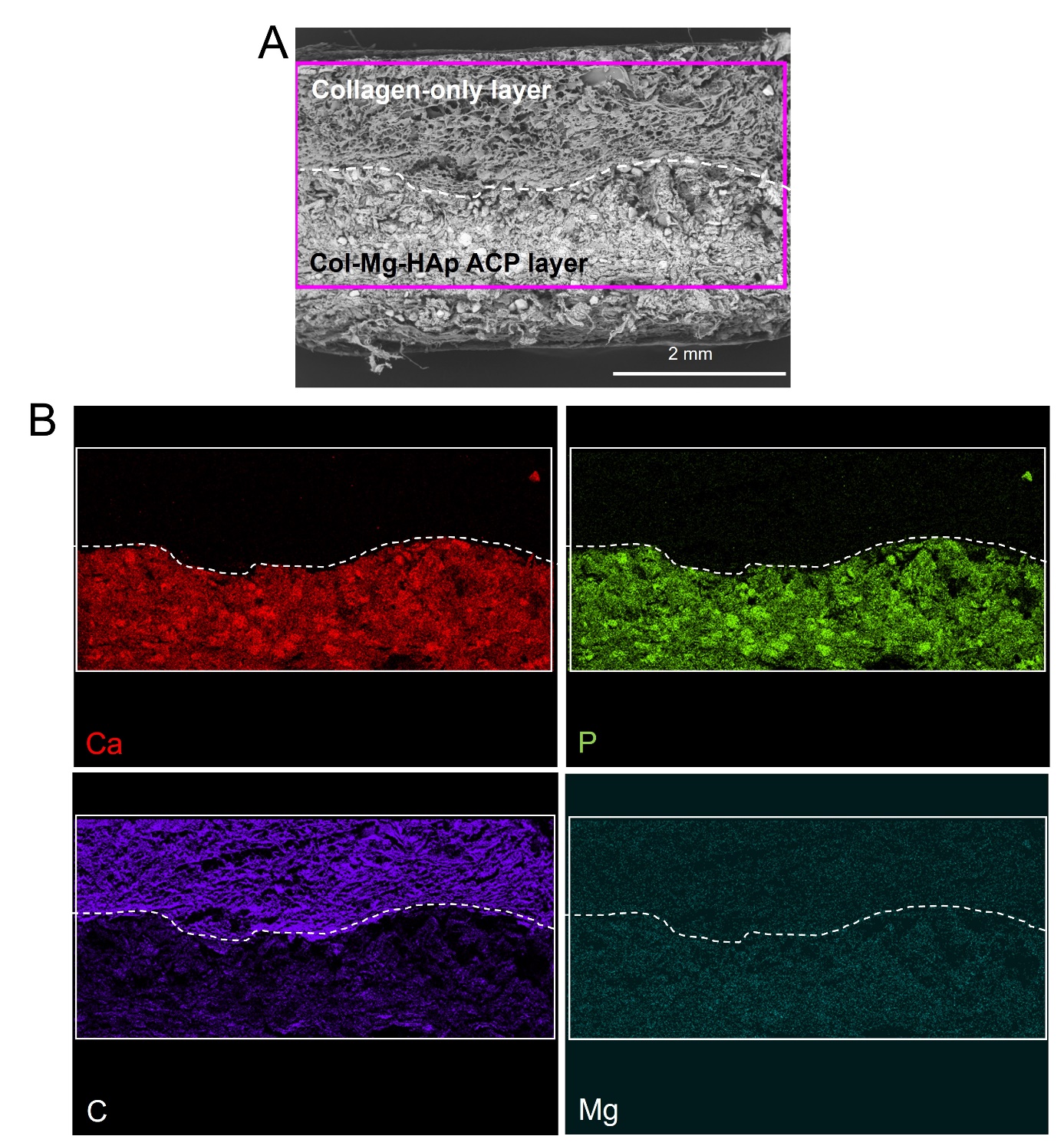


Figure S5: Morphological and chemical characterization of ACP enriched Col/Col-Mg-HAp scaffold. (A) SEM image in backscattered electron (BSE) detector mode of cross-section of the ACP granule containing Col/Col-Mg-HAp scaffold, where the top layer is collagen and the bottom layer is collagen-Mg-HAp layer enriched with ACP granules. EDS element (Calcium, Ca - red, Phosphorus, P - green, Magnesium, Mg – dark green, Carbon, C - purple) maps of the scaffold visualized on (A), where the dashed line shows the border between both layers and the brightest areas in Ca and P maps represent positions of the ACP granules.


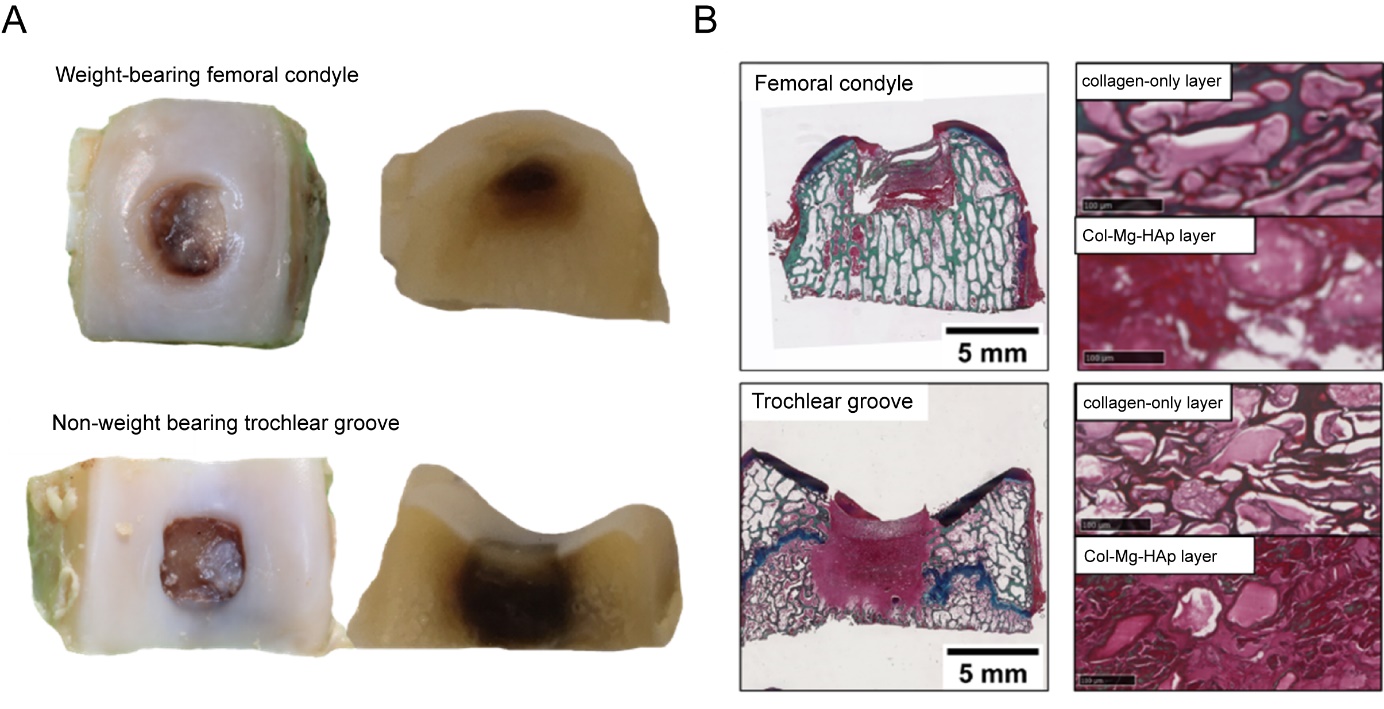


Figure S6: (A) The macroscopic appearance of a femoral condyle defect and a trochlear groove defect 2 weeks after implantation. (B) Two layers of the scaffold implanted in the femoral condyle defect after 2 weeks (stained with Alcian Blue, Fast Green, and Picrosirius Red). The scale bar indicates 5 mm and 100 µm.


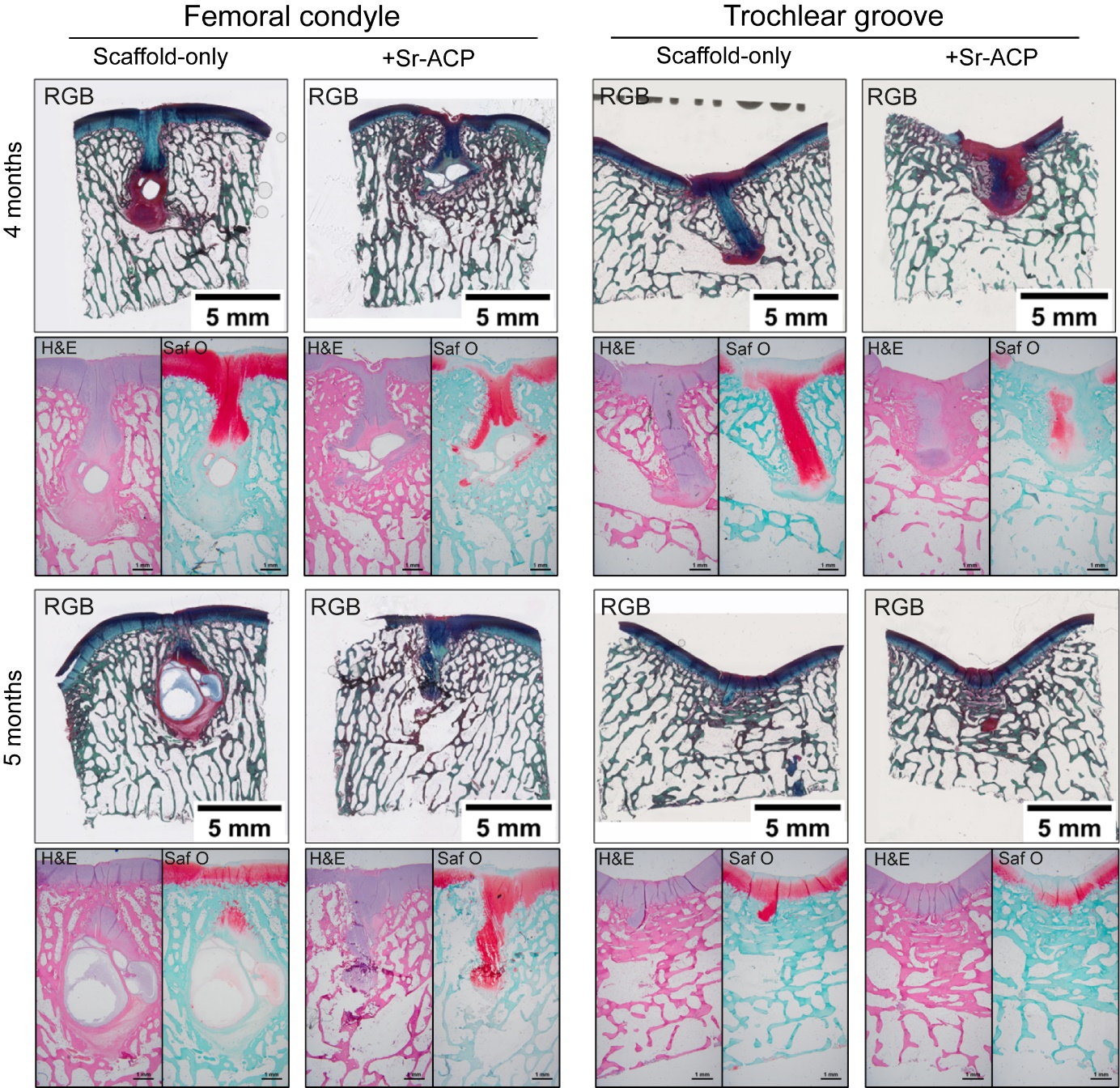


Figure S7: Osteochondral repair at 4- or 5-month post-surgery. RGB (Alcian Blue, Fast Green, and Picrosirius Red) staining, H&E staining, and Safranin-O staining of femoral condyle defects and trochlear groove defects treated with either scaffold-only or scaffold + Sr-ACP.


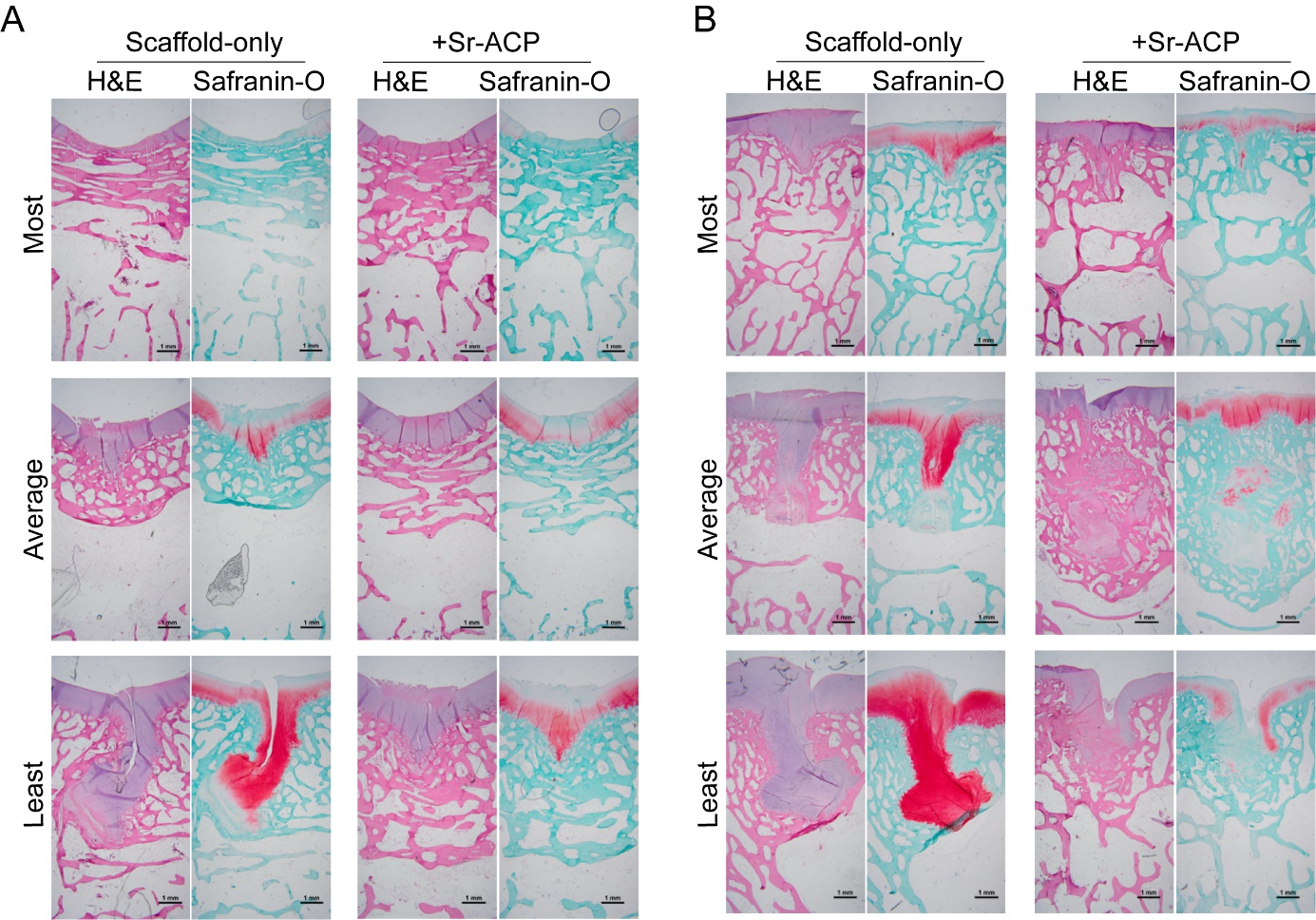
Figure S8: Osteochondral repair at 6 months post-surgery. H&E staining and Safranin-O staining of trochlear groove (A) and femoral condyle (B) defects treated with either scaffold-only or scaffold + Sr-ACP. Samples with most, average, and least bone-like tissue in bone defects are presented. The scale bar indicates 1 mm.


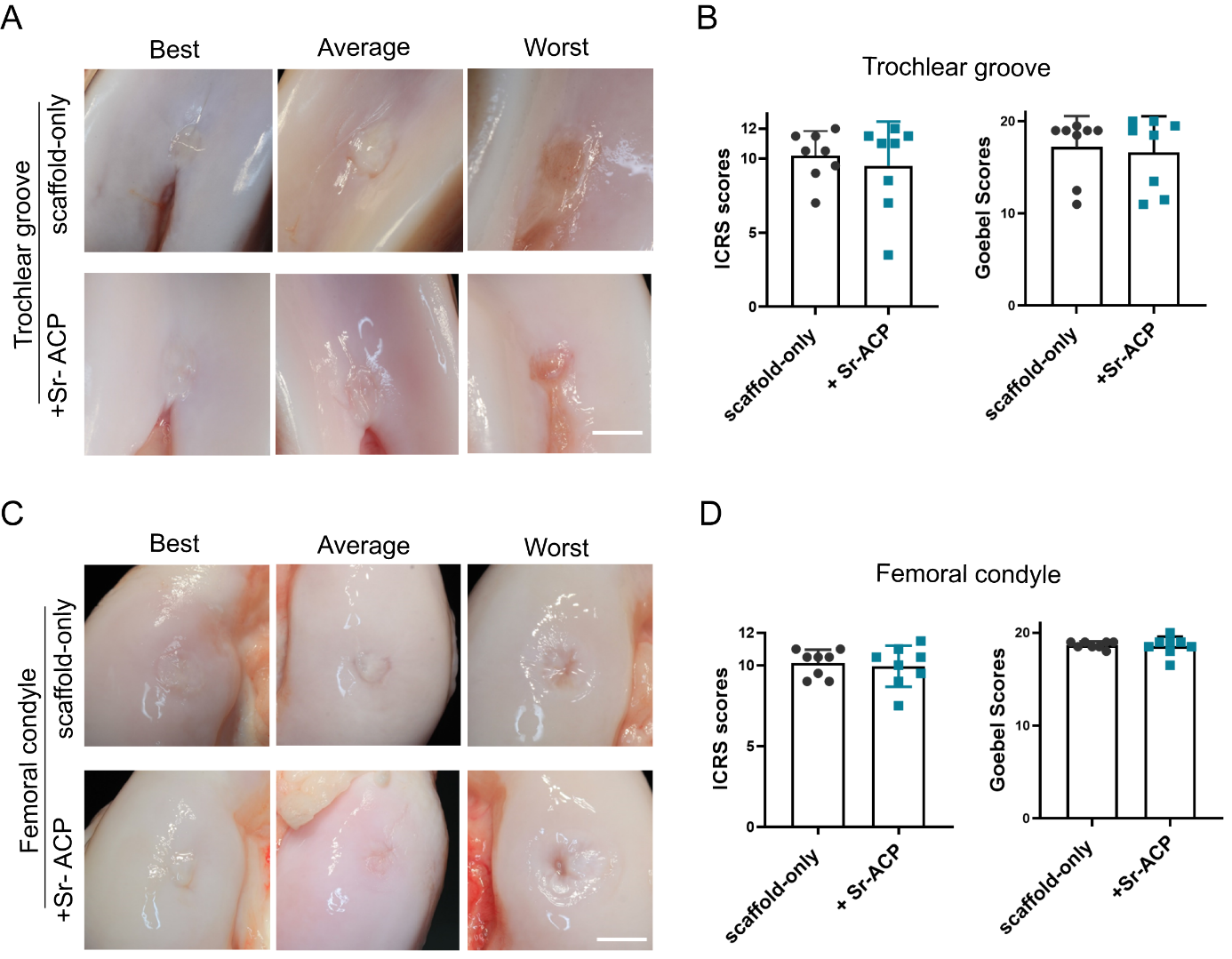


Figure S9: Macroscopic assessment of trochlear groove and femoral condyle defect repair. (A) Representative examples of trochlear groove defect sites treated with scaffold-only or Sr-ACP enriched scaffold after 6 months. Best, average, and worst samples were determined according to the ICRS scores. (B) Macroscopic scores of repair tissue in the trochlear groove defects. (C) Representative examples of femoral condyle defect sites treated with scaffold-only or scaffold with Sr-ACP after 6 months. Best, average, and worst samples were determined according to the ICRS scores. (D) Macroscopic scores of repair tissue in femoral condyle defects. The maximum score for ICRS is 12 (indicating the best), and the maximum score for Goebel score is 20 (indicating the best).
